# Association of Mental Health Disorders With Health Care Spending in the Medicare Population

**DOI:** 10.1001/jamanetworkopen.2020.1210

**Published:** 2020-03-19

**Authors:** Jose F. Figueroa, Jessica Phelan, E. John Orav, Vikram Patel, Ashish K. Jha

**Affiliations:** 1 Department of Health Policy and Management, Harvard T.H. Chan School of Public Health, Boston, Massachusetts; 2 Department of Medicine, Harvard Medical School, Boston, Massachusetts; 3 Division of General Internal Medicine, Department of Medicine, Brigham and Women’s Hospital, Boston, Massachusetts; 4 Department of Global Health and Social Medicine, Harvard Medical School, Boston, Massachusetts; 5 Harvard Global Health Institute, Harvard University, Cambridge, Massachusetts

## Abstract

**Importance:**

The degree to which the presence of mental health disorders is associated with additional medical spending on non–mental health conditions is largely unknown.

**Objective:**

To determine the proportion and degree of total spending directly associated with mental health conditions vs spending on other non–mental health conditions.

**Design, Setting, and Participants:**

This retrospective cohort study of 4 358 975 fee-for-service Medicare beneficiaries in the US in 2015 compared spending and health care utilization among Medicare patients with serious mental illness (SMI; defined as bipolar disease, schizophrenia or related psychotic disorders, and major depressive disorder), patients with other common mental health disorders (defined as anxiety disorders, personality disorders, and posttraumatic stress disorder), and patients with no known mental health disorders. Data analysis was conducted from February to October 2019.

**Exposure:**

Diagnosis of an SMI or other common mental health disorder.

**Main Outcomes and Measures:**

Risk-adjusted, standardized spending and health care utilization. Multivariable linear regression models were used to adjust for patient characteristics, including demographic characteristics and other medical comorbidities, using hospital referral region fixed effects.

**Results:**

Of 4 358 975 Medicare beneficiaries, 987 379 (22.7%) had an SMI, 326 991 (7.5%) had another common mental health disorder, and 3 044 587 (69.8%) had no known mental illness. Compared with patients with no known mental illness, patients with an SMI were younger (mean [SD] age, 72.3 [11.6] years vs 67.4 [15.7] years; *P* < .001) and more likely to have dual eligibility (633 274 [20.8%] vs 434 447 [44.0%]; *P* < .001). Patients with an SMI incurred more mean (SE) spending on mental health services than those with other common mental health disorders or no known mental illness ($2024 [3.9] vs $343 [6.2] vs $189 [2.1], respectively; *P* < .001). Patients with an SMI also had substantially higher mean (SE) spending on medical services for physical conditions than those with other common mental health disorders or no known mental illness ($17 651 [23.6] vs $15 253 [38.2] vs $12 883 [12.8], respectively; *P* < .001), reflecting $4768 (95% CI, $4713-$4823; 37% increase) more in costs for patients with an SMI and $2370 (95% CI, $2290-$2449; 18.4% increase) more in costs for patients with other common mental health disorders. Among Medicare beneficiaries, $2 686 016 110 of $64 326 262 104 total Medicare spending (4.2%) went to mental health services and an additional $5 482 791 747 (8.5%) went to additional medical spending associated with mental illness, representing a total of 12.7% of spending associated with mental health disorders.

**Conclusions and Relevance:**

In this study, having a mental health disorder was associated with spending substantially more on other medical conditions. These findings quantify the extent of additional spending in the Medicare fee-for-service population associated with a diagnosis of a mental health disorder.

## Key Points

**Question:**

What is the proportion and degree of total spending directly associated with mental health conditions compared with spending on other non–mental health conditions among Medicare patients with mental illness?

**Findings:**

In this cohort study of 4 358 975 Medicare beneficiaries, patients with serious mental illness spent substantially more on medical services for physical conditions than patients with other common mental health disorders or no known mental illness. Among Medicare beneficiaries, 4.2% of total Medicare spending went to mental health services and 8.5% went to additional medical spending associated with mental illness, for a total of 12.7% of total spending associated with mental health disorders.

**Meaning:**

In this study, having a mental health disorder was associated with substantially higher spending on for other medical conditions, which increased total spending associated with mental health disorders 3-fold.

## Teaser

This cohort study determines the proportion and degree of total spending directly associated with mental health conditions vs spending on other non–mental health conditions among Medicare beneficiaries in the US.

## Introduction

Mental health disorders are highly prevalent among the US population. In 2017, it was estimated that about 44.7 million adults, or nearly 1 in 5, have a mental health disorder, and about a quarter of those (ie, 10.4 million adults) have a serious mental illness (SMI), such as schizophrenia or bipolar disorder.^1^ Mental health disorders, beyond being a source of substantial morbidity, are also expensive to treat. In 2014 alone, the US spent $186 billion on treatment for mental health disorders, amounting to 6.4% of total US health care spending.^1^ Despite the relative financial costs of mental health disorders to the health care system, they arguably receive less general attention than other medical conditions, such as cardiovascular disease and cancer, when it comes to health care investment and funding.^2^

While direct spending on mental health disorders has been estimated to be around 6% of total US health care spending, it is not entirely clear what the true health care spending for mental health disorders is among the Medicare fee-for-service population. Mental illness has been associated with a variety of disease risk factors, such as obesity, low physical activity, and smoking.^3,4,5,6^ In addition, the presence of a mental illness can profoundly affect the ability of patients and health systems to manage other chronic medical conditions.^7^ In turn, increased risk for and poor management of chronic conditions could lead to worse health outcomes and greater use of health care services, from additional emergency department visits to hospitalizations and, with worsening progression of underlying medical conditions, more expensive interventions.^8,9^ As a result, the true financial cost of mental health disorders may be reflected in additional treatments for non–mental health conditions. While this notion is widely discussed, we are unaware of any empirical data that has, on a national scale, given an exact account of the association of mental health disorders with additional spending on medical conditions after accounting for underlying risk differences in patients with and without mental illness among the Medicare fee-for-service population. To fully understand the financial consequences of mental health disorders, it would be helpful to understand not only the amount of spending directly associated with mental illness but also the amount of spending on other conditions indirectly associated with mental illness.^10^

Therefore, using national Medicare data, we sought to answer the following 3 key questions. First, among those with mental health disorders, what proportion of total health care spending is directly spent on underlying mental health conditions vs other medical conditions? Second, is the presence of mental health disorders associated with higher spending on other medical conditions? Finally, to the degree that mental health disorders are associated with greater medical spending, what type of health care utilization is higher among patients with mental health disorders?

## Methods

This study was approved by the Harvard T.H. Chan School of Public Health institutional review board, which waived the requirement for informed consent because of the inability to contact enrollees in deidentified claims data. This study followed the Strengthening the Reporting of Observational Studies in Epidemiology (STROBE) reporting guideline.

We used a 20% sample of Medicare administrative claims data from 2015 that included Parts A, B, and D. We excluded patients with Medicare Advantage (Part C) because their claims are not available for analysis, Medicare beneficiaries without Part D because we were interested in capturing drug payments, and patients who died in the study year. As a sensitivity analysis, we repeated main analyses with decedents included.

To define mental health disorders, we used diagnoses codes identified by the Substance Abuse and Mental Health Services Administration.^11^ Following the Substance Abuse and Mental Health Services Administration, we did not define dementias, cerebral degenerations (eg, Alzheimer disease), transient mental disorders caused by conditions classified elsewhere, or intellectual disability as mental health disorders and instead classified them in the other medical condition group, given that they are considered neurological disorders. We used the *International Classification of Diseases, Ninth Revision *(*ICD*-*9*) codes for claims from January 2015 to September 2015 and then *ICD*-*10* codes from October 2015 to December 2015.

We grouped patients into 3 categories of mental illness in a waterfall fashion and, therefore, into the following mutually exclusive groups: (1) patients with SMI (ie, schizophrenia or related psychotic disorders, bipolar disease, and major depressive disorder),^12^ (2) those with other common mental health disorders (ie, anxiety disorders, personality disorders, and posttraumatic stress disorder), and (3) those with no known mental illness (ie, the remaining patients who did not have any mental health disorder). Some patients grouped in the SMI category could also have other common mental health disorders (eTable 1 in the Supplement). To identify these diagnoses, we applied the Chronic Conditions Warehouse algorithm, which was developed to help to better identify patients with chronic conditions using administrative claims data.^13^ This algorithm requires patients to have specific claims in certain care settings to classify as having a disease. We were concerned that, in some instances, the diagnosis of major depression could be the result of comorbid conditions or severity of medical illness. Therefore, as a sensitivity analysis, we excluded depression from our list of diagnoses of SMI and instead analyzed individuals with major depression separately.

To identify spending, we first calculated standardized payments for each claim based on national Medicare rates as described by the Centers for Medicare & Medicaid Services.^14^ We then categorized each claim as a mental health claim or physical medical health claim (eTable 2 in the Supplement) based on the primary diagnosis, following a similar approach used in the Substance Abuse and Mental Health Services Administration report.^11^ For example, if a patient were admitted to the hospital for a primary diagnosis of a mental illness, we would classify all payments in the inpatient setting for that hospitalization as mental health spending. For each patient, we aggregated all payments across each claim into a single number. All claims that were not classified as a mental health claim were categorized as medical spending for a physical condition. Finally, we classified the following pharmacologic classes as mental health drugs: antipsychotics, antianxiety medications, antidepressants, mood stabilizers, barbiturates, and stimulants.

Claims for substance use disorder were suppressed in Medicare claims data from 2012 to 2015.^15^ As a sensitivity analysis, we repeated our analysis using 2011 data to examine spending associated with substance use disorders.

### Statistical Analysis

We first examined differences in patient characteristics across the 3 groups. Next, we examined differences in spending associated with mental health disorders vs physical medical conditions. We used multivariable linear regression models to adjust for patient characteristics, including age, sex, dual-status eligibility, race/ethnicity, and other medical comorbidities using definitions from the Chronic Conditions Warehouse (not including diagnosis for mental health disorders). A separate regression was used with each of the following dependent variables: total spending, mental health service spending, and medical service spending. The primary factors were indicator variables for patients with an SMI and patients with other common mental health disorders. We used hospital referral region fixed effects to examine these associations within a market. We repeated separate linear regression models in patients with 1 of 5 common medical conditions, including diabetes, heart failure, chronic obstructive pulmonary disease (COPD), ischemic heart disease, and renal failure. In each model, we again adjusted for patient characteristics, as noted earlier, except for mental health disorders and the main medical condition being examined. For all models, spending for each of the 3 groups of patients was estimated by applying the model coefficients to the mean patient characteristics in the overall cohort. Finally, we examined differences in health care utilization, again using multivariable linear regression models for each measure adjusted for patient characteristics. Linear regression models were used to preserve interpretability. As a sensitivity analysis, we reran our primary models using log γ distribution, which allows for the right-skewed distribution of costs. We also ran other models in which we excluded new entrants and dual-eligible patients. All analyses were performed using SAS statistical software version 9.4 (SAS Institute). Two-tailed *t *tests were considered significant at the *P* < .05 level.

## Results

### Patient Characteristics

Our study sample included 4 358 957 Medicare beneficiaries, of whom 987 379 (22.7%) had an SMI, 326 991 (7.5%) had other common mental health disorders, and 3 044 857 (69.8%) had no known mental health disorders (Table 1). Compared with patients with no known mental illness, those diagnosed with an SMI tended to be younger (mean [SD] age, 72.3 [11.6] years vs 67.4 [15.7] years; *P* < .001), to be women (1 723 236 [56.6%] vs 667 468 [67.6%]; *P* < .001), to have black or Hispanic race/ethnicity (black: 295 325 [9.7%] vs 96 763 [9.8%]; *P* < .001; Hispanic: 60 892 [2.0%] vs 24 684 [2.5%]; *P* < .001), and to have dual eligibility (633 274 [20.8%] vs 434 447 [44.0%]; *P* < .001) (Table 1). Patients with diagnoses of other common mental health disorders were also more likely to be younger (mean [SD] age, 70.4 [13.7] years; *P* < .001) and dual-eligible (97 770 [29.9%]; *P* < .001) than those with no known mental illness. Compared with patients with other common mental illnesses or no known mental illnesses, those with an SMI had higher rates of comorbidities, including chronic kidney disease (62 455 [19.1%] vs 496 268 [16.3%] vs 247 832 [25.1%]; *P* < .001), COPD (54 607 [16.7%] vs 267 924 [8.8%] vs 200 438 [20.3%]; *P* < .001), ischemic heart disease (100 713 [30.8%] vs 788 548 [25.9%] vs 320 898 [32.5%]; *P* < .001), heart failure (51 338 [15.7%] vs 344 038 [11.3%] vs 199 451 [20.2%]; *P* < .001), and diabetes (88 615 [27.1%] vs 837 261 [25.9%] vs 335 709 [34.0%]; *P* < .001).

**Table 1.** Characteristics of Patients With Serious Mental Illness, Other Common Mental Health Disorders, and No Known Mental Illness

Patient characteristic^a^	No. (%)		
	No known mental illness (n = 3 044 587)	Other common mental health disorders (n = 326 991)^b^	Serious mental illness (n = 987 379)^c^
Age, mean (SD)	72.3 (11.6)	70.4 (13.7)	67.4 (15.7)
Women	1 723 236 (56.6)	226 278 (69.2)	667 468 (67.6)
Race/ethnicity			
Black	295 325 (9.7)	24 851 (7.6)	96 763 (9.8)
Hispanic	60 892 (2.0)	6213 (1.9)	24 684 (2.5)
White	2 517 873 (82.7)	285 136 (87.2)	832 360 (84.3)
Other^d^	170 497 (5.6)	10 791 (3.3)	33 571 (3.4)
Dual eligibility	633 274 (20.8)	97 770 (29.9)	434 447 (44.0)
Region			
Midwest	718 523 (23.6)	74 554 (22.8)	246 845 (25.0)
Northeast	605 873 (19.9)	67 687 (20.7)	203 400 (20.6)
South	1 159 988 (38.1)	135 374 (41.4)	383 103 (38.8)
West	560 204 (18.4)	49 376 (15.1)	154 031 (15.6)
Urban residence	2 706 638 (88.9)	289 060 (88.4)	878 767 (89.0)
Major comorbidities			
Chronic kidney disease	496 268 (16.3)	62 455 (19.1)	247 832 (25.1)
COPD	267 924 (8.8)	54 607 (16.7)	200 438 (20.3)
Heart failure	344 038 (11.3)	51 338 (15.7)	199 451 (20.2)
Diabetes	837 261 (27.5)	88 615 (27.1)	335 709 (34.0)
Ischemic heart disease	788 548 (25.9)	100 713 (30.8)	320 898 (32.5)
Stroke or transient ischemic attack	82 204 (2.7)	13 734 (4.2)	68 129 (6.9)

Abbreviation: COPD, chronic obstructive pulmonary disease.

^a^ All differences across gender, race/ethnicity, dual status, region, locality, and major comorbidities were statistically significant (*P* < .001).

^b^ Other common mental health disorders included anxiety disorders, personality disorders, and posttraumatic stress disorder.

^c^ Serious mental illness was defined as having schizophrenia or related psychotic disorders, bipolar disorder, or major depression.

^d^ The variable in the Medicare data describes other as Asian, Native American, and other.

### Differences in Spending

**Figure 1.**
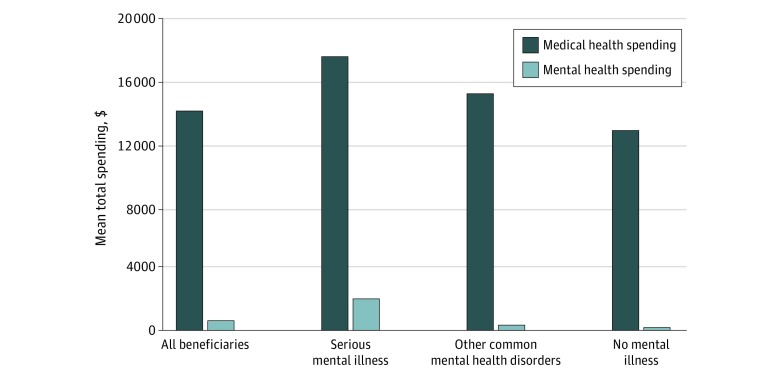
Risk-Adjusted Mean Spending Associated With Medical Services vs Mental Health Services Among Patients With and Without Mental Health Disorders

**Figure 2.**
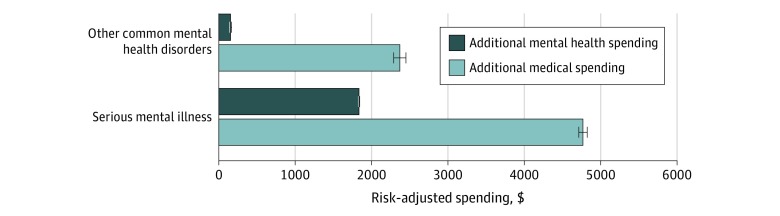
Risk-Adjusted Spending Associated With Additional Mental Health Services vs Medical Services Whiskers represent 95% CIs.

Medicare beneficiaries incurred a mean (SE) of $14 757 (12.6) per year in spending, of which a mean (SE) $616 (1.8) (4.2%) was directly associated with mental health services and a mean (SE) of $14 141 (12.4) was spent on non–mental health conditions (Figure 1). After adjustment for patient demographic characteristics and other comorbidities, patients with an SMI had higher mean (SE) spending than patients with other common mental health disorders and those with no known mental illness ($19 676 [23.8] vs $15 596 [38.6] vs $13 072 [13.0], respectively; *P* < .001) (Figure 1). Patients with an SMI had higher mean (SE) spending on mental health services than those with other common mental health disorders or patients with no known mental illness ($2024 [3.9] vs $343 [6.2] vs $189 [2.1], respectively; *P* < .001). However, patients diagnosed with an SMI or other common mental health disorder also had substantially higher mean (SE) spending on medical services for physical conditions ($17 651 [23.6] vs $15 253 [38.2] vs $12 883 [12.8], respectively) (Figure 1). Relative to patients with no known mental illness, this reflected an additional $4768 (95% CI, $4713-$4823; 37% increase) of spending for patients with an SMI and $2370 (95% CI, $2290-$2449; 18.4% increase) for patients with other common mental health disorders (Figure 2), which we defined as additional or excess medical spending associated with the presence of a mental health disorder. In the unadjusted analyses, results were similar, except these differences were generally larger (eTable 3 in the Supplement).

We found that, among Medicare beneficiaries, direct mental health spending represented approximately 4.2% (ie, $2 686 016 110 of $64 326 262 104) of total Medicare spending in the study sample. However, increased medical spending among Medicare beneficiaries with any mental health disorder represented an additional 8.5% (ie, $5 482 791 742 of $64 326 262 104) of total Medicare outlays, for a total of 12.7% of Medicare spending going either directly or indirectly to mental health (eTable 4 in the Supplement).

### Spending Among Beneficiaries With Common Chronic Conditions

We next examined spending among Medicare beneficiaries with the 5 following common chronic conditions: congestive heart failure, COPD, diabetes, chronic kidney disease, and ischemic heart disease. Patients with congestive heart failure and an SMI incurred higher mean spending than patients with other heart failure (Table 2). Relative to those with no known mental illness, patients with heart failure and an SMI incurred $9771 (95% CI, $9958-$9983) additional spending on medical health care associated with physical conditions, an increase of 32.3%. We found similar patterns for patients with an SMI and either COPD, diabetes, chronic kidney disease, or ischemic heart disease (eg, difference in total spending, COPD: $9231; 95% CI, $9026-$9436; chronic kidney disease: $10 326; 95% CI, $10 141-$10 512). In addition, patients with a major chronic condition and a common mental health disorder also incurred higher total spending related to medical health conditions than patients without a mental illness (eg, COPD, difference: $4407; 95% CI, $4113-$4702) (Table 2).

**Table 2.** Differences in Risk-Adjusted Spending Among Patients With a Major Chronic Condition and Mental Health Disorders

Spending category	Spending, mean (SE), $			Difference, mean difference (95% CI), $^a^	
	No known mental illness (n = 3 044 587)	Other common mental health disorders (n = 326 991)	Serious mental illness (n = 987 379)	Other common mental disorders vs no known mental illness	Serious mental illness vs no known mental illness
**Congestive heart failure**					
No. (%)	345 442 (11.3)	51 411 (15.7)	199 818 (20.2)	NA	NA
Total spending	30 539 (62)	36 219 (153)	41 778 (83)	5680 (5356 to 6004)	11 239 (11 026 to 11 452)
Mental health spending	283 (7)	332 (18)	1751 (10)	50 (11 to 88)	1468 (1443 to 1493)
Medical health spending	30 256 (61)	35 887 (152)	40 027 (83)	5631 (5307 to 5954)	9771 (9958 to 9983)
**COPD**					
No. (%)	266 729 (8.8)	54 527 (16.7)	200 084 (20.2)	NA	NA
Total spending	27 646 (64)	32 054 (136)	36 877 (75)	4407 (4113 to 4702)	9231 (9026 to 9436)
Mental health spending	379 (10)	370 (22)	2116 (12)	−9 (−56 to 38)	1737 (1704 to 1770)
Medical health spending	27 267 (64)	31 683 (135)	34 761 (75)	4416 (4124 to 4709)	7494 (7291 to 7697)
**Diabetes**					
No. (%)	838 542 (27.5)	88 751 (27.1)	335 487 (34.0)	NA	NA
Total spending	19 649 (31)	22 096 (92)	27 938 (51)	3257 (3065 to 3449)	8289 (8166 to 8412)
Mental health spending	238 (5)	309 (14)	2101 (8)	71 (43 to 100)	1863 (1845 to 1881)
Medical health spending	19 410 (31)	22 597 (92)	25 837 (51)	3187 (2996 to 3377)	6427 (6304 to 6549)
**Chronic kidney disease**					
No. (%)	495 133 (16.3)	62 466 (19.1)	248 171 (25.1)	NA	NA
Total spending	28 196 (51)	33 305 (138)	38 522 (74)	5109 (4819 to 5399)	10 326 (10 141 to 10 512)
Mental health spending	253 (6)	323 (17)	1941 (9)	70 (33 to 106)	1688 (1665 to 1712)
Medical health spending	27 943 (51)	32 982 (138)	36 581 (74)	5039 (4750 to 5329)	8638 (8453 to 8823)
**Ischemic heart disease**					
No. (%)	789 511 (25.9)	100 789 (30.8)	320 627 (32.5)	NA	NA
Total spending	21 896 (32)	25 619 (86)	30 986 (52)	3723 (3541 to 3904)	9090 (8964 to 9215)
Mental health spending	214 (4)	270 (12)	1683 (7)	56 (32 to 81)	1470 (1453 to 1486)
Medical health spending	21 682 (32)	25 349 (86)	29 302 (52)	3667 (3486 to 3847)	7620 (7495 to 7744)

Abbreviations: COPD, chronic obstructive pulmonary disease; NA, not applicable.

^a^ All differences in total spending, spending related to mental health services, and spending associated with medical conditions were statistically significant across the 3 groups (*P* < .001).

### Use of Specific Services Among Beneficiaries With and Without Mental Health Disorders

We examined health care utilization in acute care and nonacute care settings. Compared with patients with no known mental illness, those with an SMI had more hospitalizations in general acute care hospitals (difference, 0.136; 95% CI, 0.135-0.138; *P* < .001), days in the hospital (difference, 0.858 days; 95% CI, 0.847-0.869 days; *P* < .001), inpatient rehabilitative days (difference, 0.074 days; 95% CI, 0.071-0.076 days; *P* < .001), emergency department visits (difference, 0.496; 95% CI, 0.492-0.500; *P* < .001), observation stays (0.041; 95% CI, 0.041-0.042; *P* < .001), nursing care facility days (difference, 3.506 days; 95% CI, 3.459-3.553 days; *P* < .001), and home health service days (difference, 2.465 days; 95% CI, 2.428-2.502 days; *P* < .001). Patients with an SMI were also prescribed more total unique drugs (mean [SD], 11.30 [0.005]) relative to patients with other common mental health disorders (mean [SD], 10.09 [0.008]) and those with no known mental illness (mean [SD], 8.17 [0.003]) (difference, 3.129; 95% CI, 3.118-3.141; *P* < .001) (Table 3).

**Table 3.** Differences in Health Care Utilization of Hospital Services by Medicare Beneficiaries With and Without Mental Health Disorders

Utilization measure	No known mental illness, mean	Other common mental health disorders			Serious mental illness			*P* value
		Mean	Absolute difference (95% CI)	Increase, %	Mean	Absolute difference (95% CI)	Increase, %	
Acute care services								
Hospitalizations to general acute care hospitals, No.	0.21	0.28	0.068 (0.066 to 0.07)	33	0.35	0.136 (0.135 to 0.138)	67	<.001
Time in acute care hospitals, d	1.14	1.52	0.372 (0.356 to 0.388)	33	2.00	0.858 (0.847 to 0.869)	75	<.001
Hospitalizations to psychiatric hospitals, No.	0	0	NA	NA	0.03	NA	NA	<.001
Time in inpatient rehabilitation, d	0.05	0.07	0.019 (0.015 to 0.024)	40	0.13	0.074 (0.071 to 0.076)	160	<.001
All emergency department visits, No.	0.56	0.84	0.279 (0.273 to 0.285)	50	1.05	0.496 (0.492 to 0.500)	89	<.001
Emergency department visits with no hospitalization, No.	0.37	0.57	0.200 (0.195 to 0.205)	54	0.72	0.344 (0.341 to 0.348)	95	<.001
Observation visits with no hospitalization, No.	0.05	0.09	0.034 (0.033 to 0.035)	80	0.09	0.041 (0.041 to 0.042)	80	<.001
Nonacute care services								
Time with home health service, d	2.59	3.28	0.696 (0.642 to 0.749)	27	5.05	2.465 (2.428 to 2.502)	95	<.001
Time at nursing care facilities, d	1.97	2.14	0.179 (0.111 to 0.247)	9	5.47	3.506 (3.459 to 3.553)	178	<.001
Unique drugs, No.	8.17	10.09	1.923 (1.907 to 1.94)	24	11.3 0	3.129 (3.118 to 3.141)	38	<.001
Mental health drugs, No.	0.28	1.00	1.205 (1.190 to 1.221)	257	1.56	1.848 (1.837 to 1.859)	457	<.001
Other drugs for non–mental health conditions, No.	7.89	9.09	0.718 (0.715 to 0.721)	15	9.74	1.281 (1.279 to 1.283)	23	<.001

Abbreviation: NA, not applicable.

We also examined spending by type and care setting across the 3 groups. The largest differences in spending associated with other medical conditions between patients with an SMI and those with no known mental illness were from additional inpatient spending (mean [SE], $3491 [9.4] vs $2178 [5.1]; *P* < .001), skilled nursing facility spending (mean [SE], $1801 [5.8] vs $712 [3.1]; *P* < .001), and pharmaceutical spending (mean [SE], $4046 [12.5] vs $3308 [6.8]; *P* < .001) (eFigure 1 in the Supplement). Patients with an SMI also incurred more spending across all categories associated with direct mental health services than those with no known mental illness, including inpatient care (mean [SE], $424 [2.3] vs $36 [1.2]; *P* < .001) and mental health drugs (mean [SE], $870 [1.7] vs $95 [0.9]; *P* < .001) (eFigure 2 in the Supplement).

### Sensitivity Analyses

When we included decedents, we found that there was slightly higher mean (SE) total spending per Medicare beneficiary ($15 873 [13.0] with decedents vs $14 757 [12.6] without decedents) (eTable 5 in the Supplement). Overall, the patterns were qualitatively similar across each category. We also examined spending using 2011 data, given that claims associated with substance use disorders were suppressed in 2015. We found that very little spending was associated with substance use disorders; only a mean [SE] $31 [0.4] of $14 564 (0.2%) in total spending per Medicare beneficiary (eTable 5 in the Supplement). We also redefined SMI to exclude depression. The results were largely consistent, except that patients with bipolar disorder or schizophrenia incurred higher mean (SE) total spending than patients with major depression only ($22 025 [39.7] vs $18 535 [28.4]), associated with differences in mean (SE) spending related to mental health services ($4628 [6.2] vs $760 [4.5]) (eTable 6 in the Supplement).

We performed additional sensitivity analyses in which we looked back across 2 years of data to identify mental health diagnoses. The results were qualitatively similar with the analysis in which we used 1 year of data (eTable 7 in the Supplement). Excluding new entrants and excluding dual-eligible patients led to similar results as well (eTable 7 in the Supplement). Finally, because health care costs are not normally distributive, we also reran our primary models using a log γ distribution, and our results were also consistent with our linear models (eTable 8 in the Supplement).

## Discussion

In the Medicare population, we found substantial differences in spending among patients with a diagnosis of an SMI or other common mental health disorder compared with patients with no known mental illness. Patients with mental health disorders incurred higher spending on mental health services than patients with no mental disorders. However, we found substantial differences in spending associated with non–mental health conditions, even after adjusting for underlying differences in patient demographic characteristics and clinical risk. Compared with those with no known mental illness, patients with an SMI had increased spending on non–mental health conditions by more than one-third (nearly $5000), and the presence of other common mental health disorders, like anxiety or personality disorders, was associated with increased spending on non–mental health spending by nearly 20% (approximately $2400). While we found that direct spending on mental health services for the Medicare population was 4.2% of total health care spending, we estimated an additional 8.5% of total Medicare spending is because of additional spending on non–mental health conditions among beneficiaries with mental health disorders. Taken together, these findings suggest that we are substantially underestimating the full health care spending on mental illness, with the additional amount of spending on medical conditions being twice as high as the direct mental health spending.

Our findings confirm that spending associated with mental health services is much greater than is generally reported. Prior national estimates on the amount of total health care spending associated with mental health disorders has been about 6.4% in the general population,^11^ which includes a healthier and younger population than our sample. Among the Medicare population, the lower estimate of 4.2% of total spending on mental health services likely reflects higher spending on physical conditions, such as diabetes and heart failure. Additionally, we found that only approximately $2000 per year represented direct spending on mental health services among Medicare beneficiaries with serious mental illness. This may be for several reasons. First, we included major depression as an SMI, but depression is associated with much lower spending for direct mental health services than schizophrenia and bipolar disorder, which are closer to $5000 per person, as shown in our sensitivity analysis. Also, the mean age of individuals in the subgroup with an SMI was older than 65 years, suggesting that many of these patients likely have had their diagnosis for decades, given that most patients with schizophrenia and bipolar disorder are diagnosed in early adulthood.^16^ Therefore, it is possible that Medicare patients aged 65 years and older with schizophrenia or bipolar disease have milder versions of these conditions than other younger patients with an SMI, many of whom may experience an early death.^17,18^ Younger patients with an SMI may also be covered by a different insurance program (eg, Medicaid) and therefore are not reflected in our sample.^19,20^ Differences in what insurances cover (ie, the difference between commercial insurance and Medicaid) may also explain this finding. Finally, prior work has found that older adults are much less likely to use behavioral health services than younger patients.^21^

However, as hypothesized, we still found a 3-fold increase in spending associated with mental health disorders when accounting for how they are associated with increased spending on other non–mental health conditions. It is likely that mental illness impairs the ability of patients and health systems to take effective care of chronic medical conditions. People with mental health disorders are more likely to develop chronic conditions, and their progression of disease is often much worse.^22^ For example, patients with depression and cardiovascular disease have lower rates of medication adherence, increased rates of smoking, and lower levels of physical activity, all of which contribute to worse cardiac outcomes.^23,24^ In addition, people with mental health disorders may be less likely to receive life-saving therapies.^25^ The combination of these factors may contribute to worse health outcomes and higher spending, much of which may have been preventable with effective management of patients’ mental illness.

These data suggest that interventions that effectively treat mental health disorders among patients with chronic conditions may lead to substantial reductions in total spending. To achieve this, greater integration of primary care and mental health care may be an important strategy.^26,27^ A 2013 estimate^28^ suggested that approximately $52 billion annually could be saved with better integration of mental and behavioral health treatments with medical treatments, perhaps through payment models that support shared responsibility among mental health providers and other medical care providers. Importantly, such efforts can also lead to concomitant improvement in quality of care delivered to these at-risk populations.

Of note, we found a higher rate of SMI in the Medicare population than in the general population. This could be because the prevalence of major depression is much higher in an elderly population with high levels of comorbidities than in a younger, healthier population.^29^ Also, our sample included dual-eligible patients, who have much higher rates of SMI compared with the general population.^30^

We also found some spending among patients with no known mental health disorders that was classified as mental health spending, much of which was related to mental health drugs. It is possible that this could reflect prescriptions of psychiatric medications to treat non–mental health conditions, such as insomnia, nausea, or even chronic pain.^31^ However, it is also possible that some of these patients were not identified as having a mental health disorder by administrative claim diagnoses when they actually do have a mental illness. To the extent that the latter is the case, this suggests that spending associated with mental illness may potentially be higher.

Our work adds to an existing body of literature on the association of mental health disorders with health spending. Several prior studies^12,21,32^ have described higher overall spending among patients with mental health disorders. Our findings extend this work by using national data and risk adjustment to account for underlying differences in patient populations. While no risk adjustment is perfect, our approach helped to produce a more accurate estimate of the additional spending associated with mental health disorders. Freeman et al^33^ also found that, in a Medicaid population, most spending among patients with mental illness was because of spending on non–mental health conditions. However, Medicaid populations vary widely across states and may not be generalizable to the broader, older population. Cloutier et al^20,34^ also examined spending associated with schizophrenia and bipolar disorder, although these studies were national aggregated estimates that did not specifically examine health care utilization. Thorpe et al^32^ similarly reported the association between increased numbers of physical comorbidities and increased spending associated with behavioral health and, in particular, depression. Likewise, other work found increased spending because of chronic illnesses in patients with depression, albeit in a small sample of privately insured patients across a few practices.^35,36^ Our approach began with a focus on mental illness more broadly and was applied to Medicare patients nationally.

### Limitations

Our work has several limitations. First, we used administrative claims data to identify comorbidities and were thus unable to identify patients with mental health disorders that severely affect functional capacity vs those that do not. It was also not possible to determine whether any specific diseases were in remission, which introduces some misclassification that would bias us to the null hypothesis. Next, our sample only included Medicare fee-for-service patients, which limited the generalizability of our results. Our analysis also included patients who were dually eligible for Medicare and Medicaid, of whom a large proportion had mental health disorders. Importantly, the Medicare administrative claims data does not capture spending incurred under Medicaid, and therefore, we are likely underestimating the full amount of spending of patients with dual eligibility, especially spending associated with long-term care. Also, our results did not account for indirect losses of revenue stemming from lower productivity, missed days of work, or nonhealth direct costs associated with law enforcement, homeless shelters, and research and training. Prior work has shown that these costs among patients with an SMI are substantial,^20,34^ and accounting for such costs would be important to understand the full nonmedical costs associated with mental health disorders.

## Conclusions

In this study, the presence of an SMI or other common mental health disorders was associated with substantially higher spending for non–mental health conditions. Additionally, excess spending on non–mental health conditions was twice as large as spending on mental health disorders. These findings suggest that nearly 13% of all Medicare spending may be associated with mental health disorders, a 3-fold increase compared with spending directly associated with mental health services. Our analyses highlight the importance of managing mental health disorders to improve quality of care and decrease spending across a range of conditions for patients with mental illness.

## Notes

Supplement. **eTable 1. **Study Sample of Medicare Beneficiaries **eTable 2. **Types of Claims Classified as Mental Health Services **eTable 3. **Mean Spending per Medicare Beneficiary With Mental Health Disorders, Unadjusted Results **eTable 4. **Increased Medical Spending Associated With the Presence of Mental Health Disorders **eTable 5. **Mean Spending per Medicare Beneficiary in 2011 and 2015 With and Without Decedents **eTable 6. **Differences in Risk-Adjusted Spending in Patients With Serious Mental Illness (Excluding Depression), Major Depression, Other Common Mental Health Disorders, and With No Mental Illness **eTable 7. **Additional Sensitivity Analyses With Different Inclusion and Exclusion Criteria for Decedents, New Entrants, and Dual-Eligible Beneficiaries **eTable 8. **Log Gamma Distribution Model **eFigure 1. **Risk-Adjusted Spending Associated With Medical Conditions in Patients With and Without Mental Health Disorders **eFigure 2. **Differences in Risk-Adjusted Spending Associated With Mental Health Services in Patients With and Without Mental Health Disorders
