# Supplementary material for: Association of Mental Health Disorders With Health Care Spending in the Medicare Population
Source: JAMA Netw Open. 2020 Mar 19;3(3):e201210. doi: 10.1001/jamanetworkopen.2020.1210 (PMC7082719; doi:10.1001/jamanetworkopen.2020.1210)
Supplement: Supplement. — eTable 1. Study Sample of Medicare Beneficiaries eTable 2. Types of Claims Classified as Mental Health Services eTable 3. Mean Spending per Medicare Beneficiary With Mental Health Disorders, Unadjusted Results eTable 4. Increased Medical Spending Associated With the Presence of Mental Health Disorders eTable 5. Mean Spending per Medicare Beneficiary in 2011 and 2015 With and Without Decedents eTable 6. Differences in Risk-Adjusted Spending in Patients With Serious Mental Illness (Excluding Depression), Major Depression, Other Common Mental Health Disorders, and With No Mental Illness eTable 7. Additional Sensitivity Analyses With Different Inclusion and Exclusion Criteria for Decedents, New Entrants, and Dual-Eligible Beneficiaries eTable 8. Log Gamma Distribution Model eFigure 1. Risk-Adjusted Spending Associated With Medical Conditions in Patients With and Without Mental Health Disorders eFigure 2. Differences in Risk-Adjusted Spending Associated With Mental Health Services in Patients With and Without Mental Health Disorders [file jamanetwopen-3-e201210-s001.pdf]

# Supplementary Online Content

Figueroa JF, Phelan J, Orav EJ, Patel V, Jha AK. Association of mental health disorders with health care spending in the Medicare population. *JAMA Netw Open*. 2020;3(3):e201210. doi:10.1001/jamanetworkopen.2020.1210

**eTable 1.** Study Sample of Medicare Beneficiaries
**eTable 2.** Types of Claims Classified as Mental Health Services
**eTable 3.** Mean Spending per Medicare Beneficiary With Mental Health Disorders, Unadjusted Results
**eTable 4.** Increased Medical Spending Associated With the Presence of Mental Health Disorders
**eTable 5.** Mean Spending per Medicare Beneficiary in 2011 and 2015 With and Without Decedents
**eTable 6.** Differences in Risk-Adjusted Spending in Patients with Serious Mental Illness (Excluding Depression), Major Depression, Other Common Mental Health Disorders, and With No Mental Illness
**eTable 7.** Additional Sensitivity Analyses With Different Inclusion and Exclusion Criteria for Decedents, New Entrants, and Dual-Eligible Beneficiaries
**eTable 8.** Log Gamma Distribution Model
**eFigure 1.** Risk-Adjusted Spending Associated With Medical Conditions in Patients With and Without Mental Health Disorders
**eFigure 2.** Differences in Risk-Adjusted Spending Associated With Mental Health Services in Patients With and Without Mental Health Disorders

This supplementary material has been provided by the authors to give readers additional information about their work.

© 2020 Figueroa JF et al. *JAMA Network Open.*

**eTable 1.** Study Sample of Medicare Beneficiaries

| Medicare Sample | Serious Mental Illness | Other Common Mental Health Disorders | No Known Mental Illness |
|---|---|---|---|
| Total no. of beneficiaries with Part D | 1,073,716 | 346,098 | 3,160,789 |
| Excluding decedents | 987,379 | 326,991 | 3,044,587 |
| Excluding new entrants and decedents | 973,910 | 320,335 | 2,931,786 |
| Excluding decedents and dual-eligible patients | 552,441 | 229,264 | 2,412,153 |

© 2020 Figueroa JF et al. *JAMA Network Open.*

**eTable 2.** Types of Claims Classified as Mental Health Services

| | |
|---|---|
| **Inpatient File** | All admissions to acute care hospitals and critical access hospitals with a primary diagnosis for a mental health disorder or substance abuse disorder: ICD 9 codes of 239-319 and ICD 10 codes F00-F99 (starting October 2015)<br><br>All admissions with a primary diagnosis of intentional self-harm (E950-952 and X60-X84)<br><br>All admissions regardless of primary diagnosis to psychiatric hospitals. |
| **Outpatient File, Skilled Nursing Facility File, Home Health File, Durable Medical Equipment File, and Hospice File** | All claims in these files with the primary diagnosis of a mental health disorder or substance abuse disorder (same diagnosis codes as above)<br><br>All claims in these files with the primary diagnosis of intentional self-harm (same diagnosis codes as above) |
| **Claims from Mental Health Providers in Carrier and Outpatient File** | All claims from providers with the following NPI specialty codes: psychiatry (26) , general psychiatry (27), clinical psychologist (78), and neuropsychiatry (86) |
| **Procedures/tests in Outpatient and Carrier File** | All billing codes related to psychiatric diagnostic evaluations, psychotherapy, psychological testing, and electroconvulsive therapy.<br><br>CPT codes include: 90791, 90792, 90832, 90833, 90834, 90836, 90837, 90838, 90839, 90846, 90847, 90849, 90853, 90870, 96101, 96118. |
| **Drugs in Part D File** | All drugs in the following pharmacologic classes were defined as mental health drugs: antipsychotics, anti-anxiety medications, anti-depressants, barbiturates, mood stabilizers, and stimulants. |

We used a similar approach for defining claims as mental health claims as has been previously done by SAMHSA. For the inpatient file, we categorized all admissions for a primary diagnosis of a mental health disorder or intentional self-harm as a mental health claim. All admissions, regardless of primary diagnosis, to psychiatric hospitals were also included as mental health claims. We also categorized each claim in the outpatient file, skilled nursing facility file, home health service file, and hospice file as a mental health claim if the primary diagnosis was for a mental health disorder or intentional self-harm. Next, we categorized all claims as mental health related if filed by provider with a NPI specialty code of psychiatry, general psychiatry, clinical psychologist, and neuropsychiatry. We also categorized the following set of billing codes as mental health claims: psychiatric diagnostic evaluations, psychotherapy, psychological testing, and electroconvulsive therapy as mental health claims.

© 2020 Figueroa JF et al. *JAMA Network Open.*

**eTable 3.** Mean Spending per Medicare Beneficiary With Mental Health Disorders, Unadjusted Results

| | **Serious Mental Illness** N=3,044,587 | **Other Common Mental Health Disorders** N=326,991 | **No Known Mental Illness** N=987,379 |
|---|---|---|---|
| **Total Spending** | $26,122 | $17,628 | $10,763 |
| Mental Health | $2,369 | $386 | $72 |
| Medical Health | $23,752 | $17,242 | $10,690 |
| **Inpatient Spending** | $5,735 | $3,486 | $1,565 |
| Mental Health | $524 | $13 | $1 |
| Medical Health | $5,210 | $3,473 | $1,564 |
| **Outpatient Spending** | $3,901 | $3,122 | $2,070 |
| Mental Health | $269 | $34 | $5 |
| Medical | $3,632 | $3,088 | $2,065 |
| **Physician Services** | $2,361 | $1,684 | $1,004 |
| Mental Health | $404 | $93 | $7 |
| Medical | $1,957 | $1,591 | $997 |
| **Tests/Procedures** | $1,940 | $1,897 | $1,388 |
| Mental Health | $30 | $10 | $2 |
| Medical | $1,911 | $1,887 | $1,386 |
| **Skilled Nursing Facility** | $2,800 | $1,028 | $394 |
| Mental Health | $77 | $9 | $2 |
| Medical | $2,723 | $1,019 | $391 |
| **Home Health Services** | $1,165 | $636 | $300 |
| Mental Health | $35 | $5 | $1 |
| Medical | $1,130 | $631 | $298 |
| **Hospice** | $427 | $269 | $128 |
| Mental Health | $1 | $0 | $0 |
| Medical | $427 | $269 | $128 |
| **Drugs (Part D)** | $6,162 | $4,177 | $2,965 |
| Mental Health | $996 | $217 | $53 |
| Medical | $5,166 | $3,960 | $2,913 |

**eTable 4.** Increased Medical Spending Associated With the Presence of Mental Health Disorders

| Medicare Beneficiaries | No. of Patients | Mean total spending | Total Spending | Total Medical Spending | Total Mental Health Spending | Excess Medical Spending | Total Spending Related to Mental Health |
|---|---|---|---|---|---|---|---|
| **Serious Mental Illness** | 987,379 | $19,676 | $19,427,669,204 | $17,428,226,729 | $1,998,553,834 | $4,707,823,072 | $6,706,376,906 |
| **Other Common Mental Health Disorders** | 326,991 | $15,596 | $5,099,751,636 | $4,987,593,723 | $112,278,900 | $774,968,670 | $887,247,570 |
| **No Known Mental Illness** | 3,044,587 | $13,072 | $39,798,841,264 | $39,223,414,321 | $575,183,376 | 0 | $575,183,376 |
| **All Beneficiaries** | 4,358,957 | $48,344 | $64,326,262,104 | $61,639,234,773 | $2,686,016,110 | $5,482,791,742 | $8,168,807,852 |
| | | | | **% of Total:** | 4.2% | 8.5% | 12.7% |

© 2020 Figueroa JF et al. *JAMA Network Open.*

**eTable 5.** Mean Spending per Medicare Beneficiary in 2011 and 2015 With and Without Decedents

| | 2011 | | 2015 | |
|---|---|---|---|---|
| | **Excluding Decedents** | **Including Decedents** | **Excluding Decedents** | **Including Decedents** |
| **Total Spending** | $14,564 | $15,780 | $14,757 | $15,873 |
| Mental Health | $950 | $965 | $616 | $620 |
| Substance Abuse | $31 | $31 | $0 | $0 |
| Medical Health | $13,583 | $14,785 | $14,140 | $15,253 |
| **Inpatient Spending** | $3,141 | $3,850 | $2,654 | $3,274 |
| Mental Health | $207 | $209 | $121 | $127 |
| Substance Abuse | $19 | $19 | $0 | $0 |
| Medical Health | $2,915 | $3,622 | $2,533 | $3,147 |
| **Outpatient Spending** | $2,235 | $2,263 | $2,564 | $2,605 |
| Mental Health | $77 | $76 | $67 | $67 |
| Substance Abuse | $4 | $4 | $0 | $0 |
| Medical | $2,153 | $2,183 | $2,497 | $2,538 |
| **Physician Services & Tests** | $3,563 | $3,706 | $3,658 | $3,783 |
| Mental Health | $126 | $126 | $120 | $121 |
| Substance Abuse | $7 | $7 | $0 | $0 |
| Medical | $3,430 | $3,573 | $3,537 | $3,662 |
| **Skilled Nursing Facility** | $1,112 | $1,308 | $986 | $1,166 |
| Mental Health | $28 | $33 | $20 | $23 |
| Substance Abuse | $1 | $1 | $0 | $0 |
| Medical | $1,083 | $1,274 | $967 | $1,143 |
| **Home Health Services** | $620 | $653 | $521 | $554 |
| Mental Health | $12 | $12 | $9 | $10 |
| Substance Abuse | $0 | $0 | $0 | $0 |
| Medical | $608 | $641 | $512 | $544 |
| **Hospice** | $204 | $357 | $207 | $374 |
| Mental Health | $36 | $55 | $0 | $0 |
| Substance Abuse | $0 | $0 | $0 | $0 |
| Medical | $168 | $302 | $206 | $374 |
| **Drugs (Part D)** | $3,288 | $3,235 | $3,780 | $3,724 |
| Mental Health | $463 | $452 | $279 | $271 |
| Medical | $2,825 | $2,783 | $3,502 | $3,454 |

© 2020 Figueroa JF et al. *JAMA Network Open.*

**eTable 6.** Differences in Risk-Adjusted Spending in Patients with Serious Mental Illness (Excluding Depression), Major Depression, Other Common Mental Health Disorders, and With No Mental Illness

| | Serious Mental Illness Redefined as Schizophrenia/Related Psychotic Diseases or Bipolar Disorder | Major Depression | Other Common Mental Health Disorders | No Known Mental Illness |
|---|---|---|---|---|
| **Total Spending** | $22,025 | $18,535 | $15,597 | $13,042 |
| Mental Health | $4,628 | $760 | $345 | $155 |
| Medical Health | $17,397 | $17,775 | $15,252 | $12,886 |
| **Inpatient Spending** | $4,966 | $3,404 | $2,861 | $2,209 |
| Mental Health | $1,265 | $15 | -$2 | $25 |
| Medical Health | $3,701 | $3,388 | $2,863 | $2,184 |
| **Outpatient Spending** | $2,814 | $3,030 | $2,820 | $2,410 |
| Mental Health | $531 | $89 | $31 | $13 |
| Medical | $2,282 | $2,941 | $2,788 | $2,397 |
| **Physician Services** | $2,303 | $1,731 | $1,509 | $1,161 |
| Mental Health | $647 | $229 | $89 | $16 |
| Medical | $1,656 | $1,501 | $1,420 | $1,145 |
| **Tests/Procedures** | $1,518 | $1,790 | $1,731 | $1,486 |
| Mental Health | $42 | $18 | $10 | $3 |
| Medical | $1,477 | $1,772 | $1,721 | $1,483 |
| **Skilled Nursing Facility** | $2,765 | $1,410 | $822 | $712 |
| Mental Health | $138 | $9 | $6 | $10 |
| Medical | $2,626 | $1,401 | $816 | $702 |
| **Home Health Services** | $782 | $847 | $533 | $422 |
| Mental Health | $52 | $16 | $4 | $4 |
| Medical | $731 | $831 | $530 | $418 |
| **Hospice** | $366 | $291 | $285 | $162 |
| Mental Health | $1 | $0 | $0 | $0 |
| Medical | $365 | $291 | $285 | $162 |
| **Drugs (Part D)** | $5,328 | $4,716 | $3,865 | $3,398 |
| Mental Health | $1,882 | $378 | $202 | $82 |
| Medical | $3,445 | $4,338 | $3,663 | $3,316 |

© 2020 Figueroa JF et al. *JAMA Network Open.*

**eTable 7.** Additional Sensitivity Analyses With Different Inclusion and Exclusion Criteria for Decedents, New Entrants, and Dual-Eligible Beneficiaries

| | No Known Mental Illness | Other Common Mental Health Disorders | Serious Mental Illness | Difference: Other Common Mental Disorders vs. No Known Mental Illness | Difference: Serious Mental Illness vs. No Known Mental Illness |
|---|---|---|---|---|---|
| **Original analyses (excludes decedents; allows new entrants)** | | | | | |
| Mean Total Spending | $13,072 | $15,596 | $19,675 | $2,524 [$2,444 to $2,604] | $6,603 [$6,548 to $6,659] |
| Mental Health | $189 | $343 | $2,024 | $154 [$141 to $167] | $1,835 [$1,826 to $1,844] |
| Medical Health | $12,883 | $15,253 | $17,651 | $2,369 [$2,290 to $2,449] | $4,768 [$4,713 to $4,823] |
| **Using 2-years of data to classify people with mental illness** | | | | | |
| Mean Total Spending | $13,145 | $14,912 | $18,374 | $1,767 [$1,691 to $1,843] | $5,230 [$5,177 to $5,282] |
| Mental Health | $200 | $281 | $1,666 | $82 [$69 to $94] | $1,466 [$1,457 to $1,474] |
| Medical Health | $12,945 | $14,630 | $16,708 | $1,686 [$1,610 to $1,761] | $3,764 [$3,712 to $3,815] |
| **Analyses excluding decedents and no new entrants** | | | | | |
| Mean Total Spending | $14,157 | $16,068 | $19,490 | $1,912 [$1,834 to $1,989] | $5,333 [$5,280 to $5,386] |
| Mental Health | $200 | $283 | $1,617 | $82 [$70 to $95] | $1,417 [$1,408 to $1,425] |
| Medical Health | $13,956 | $15,786 | $17,872 | $1,829 [$1,752 to $1,906] | $3,916 [$3,863 to $3,968] |
| **Analyses including both decedents and new entrants** | | | | | |
| Mean Total Spending | $14,447 | $16,355 | $19,775 | $1,907 [$1,828 to $1,987] | $5,328 [$5,274 to $5,382] |
| Mental Health | $206 | $286 | $1,627 | $80 [$67 to $92] | $1,421 [$1,412 to $1,429] |
| Medical Health | $14,241 | $16,069 | $18,148 | $1,828 [$1,749 to $1,906] | $3,908 [$3,854 to $3,961] |
| **Analyses excluding dual-eligible patients** | | | | | |
| Mean Total Spending | $11,774 | $13,644 | $16,905 | $1,871 [$1,790 to $1,951] | $5,131 [$5,073 to $5,189] |
| Mental Health | $86 | $193 | $986 | $107 [$98 to $116] | $900 [$893 to $906] |
| Medical Health | $11,688 | $13,451 | $15,919 | $1,763 [$1,684 to $1,843] | $4,231 [$4,174 to $4,289] |

© 2020 Figueroa JF et al. *JAMA Network Open.*

**eTable 8.** Log Gamma Distribution Model

| | **Difference between those with other common mental health disorders vs. those with no known mental illness** | | | **Difference between people with SMI vs. those with no known mental illness** | | |
|---|---|---|---|---|---|---|
| | **Model parameter**: People with common mental health disorders vs. those with no known mental illness | **% relative difference** | **p-value** | **Model Parameter:** People with SMI vs. those with no known mental illness | **% relative difference** | **p-value** |
| Mean Total Spending | 0.23 | 26.7% | <0.001 | 0.42 | 52.3% | <0.001 |
| Mental Health | 1.59 | 390.4% | <0.001 | 2.98 | 1871.3% | <0.001 |
| Medical Health | 0.31 | 24.5% | <0.001 | 0.31 | 36.6% | <0.001 |

© 2020 Figueroa JF et al. *JAMA Network Open.*

**eFigure 1.** Risk-Adjusted Spending Associated With Medical Conditions in Patients With and Without Mental Health Disorders

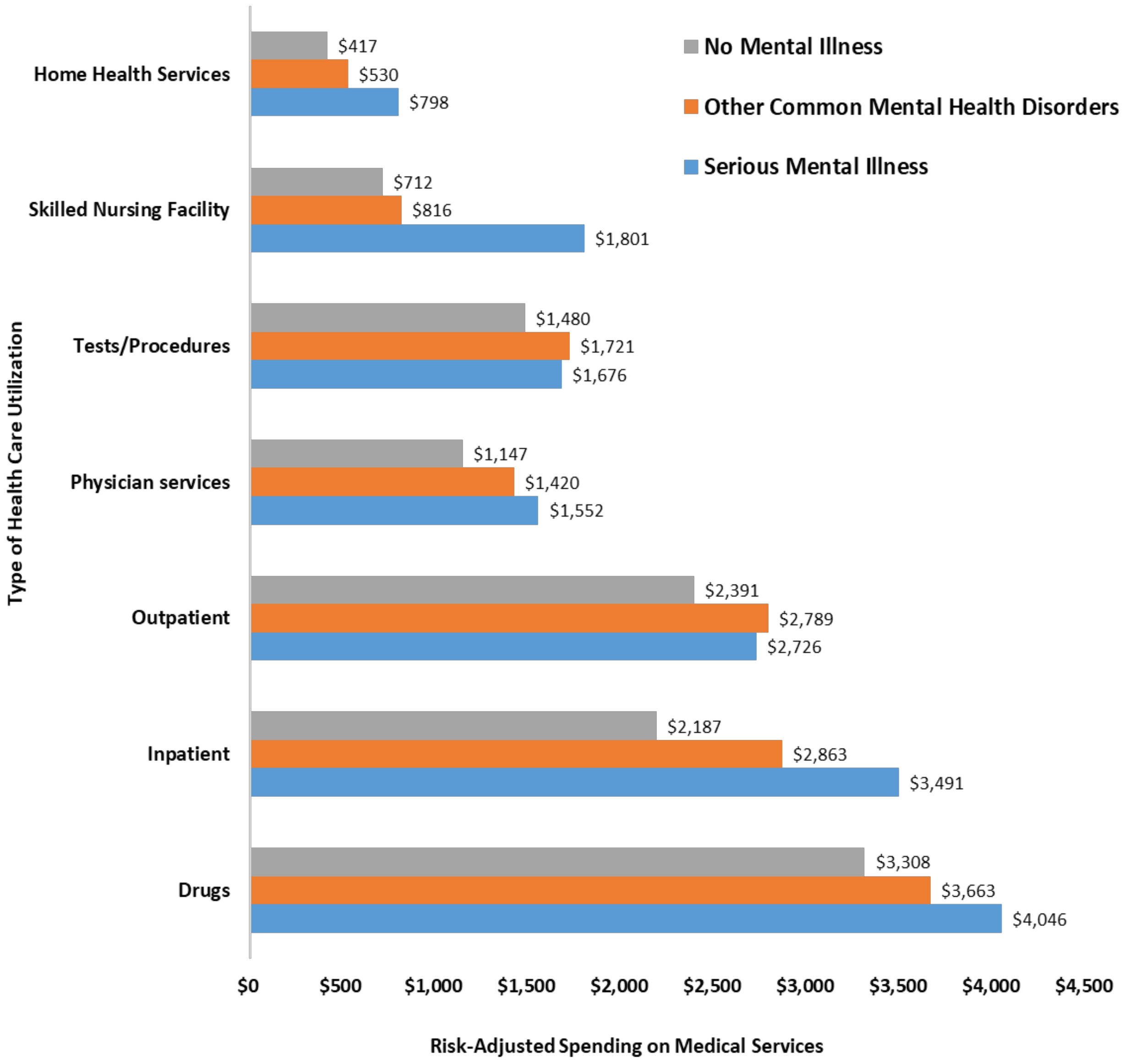


© 2020 Figueroa JF et al. *JAMA Network Open.*

**eFigure 2.** Differences in Risk-Adjusted Spending Associated With Mental Health Services in Patients With and Without Mental Health Disorders

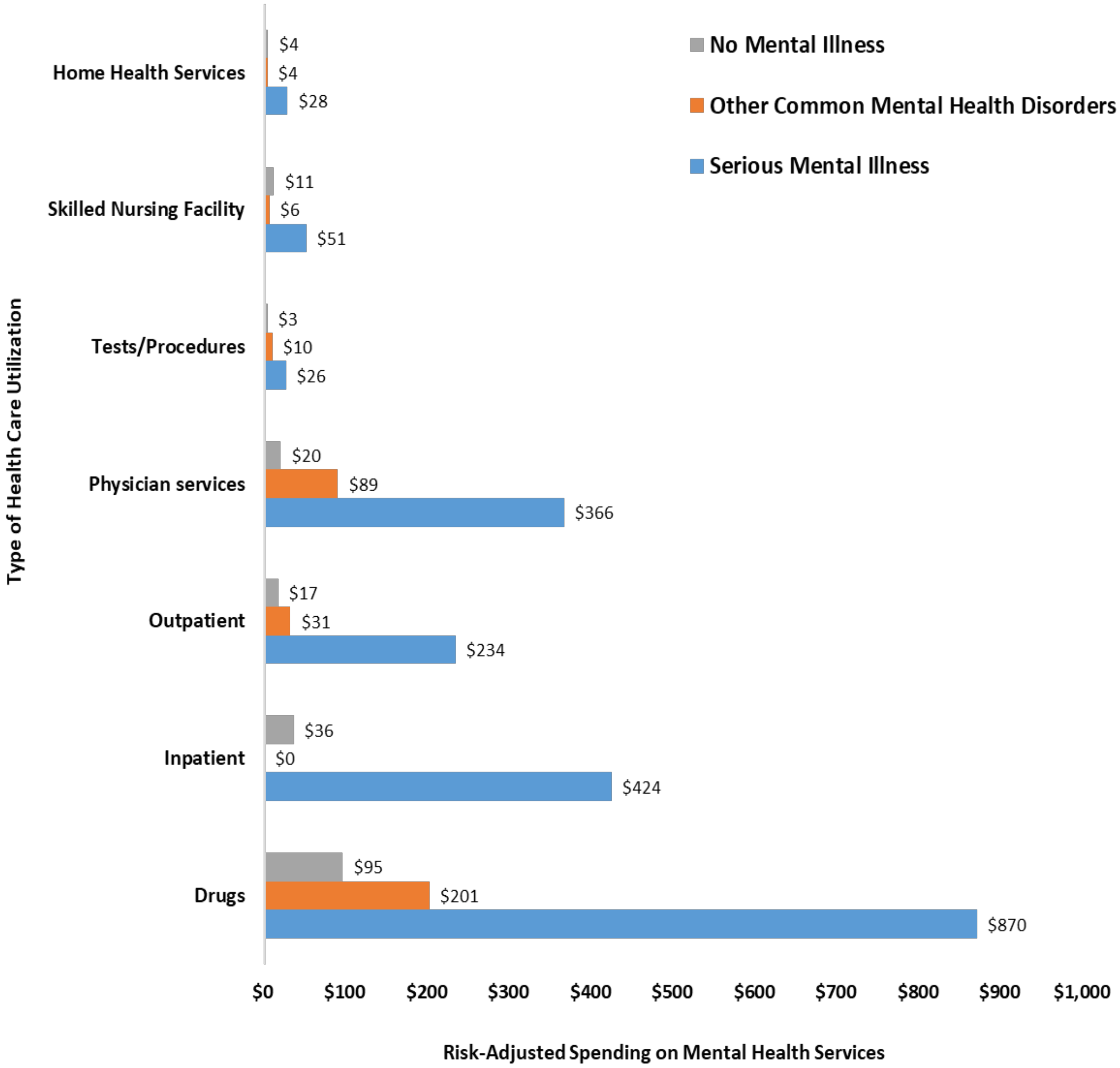


© 2020 Figueroa JF et al. *JAMA Network Open.*
